# Occupational and Personal Determinants of Musculoskeletal Disorders among Urban Taxi Drivers in Ghana

**DOI:** 10.1155/2014/517259

**Published:** 2014-08-14

**Authors:** J. K. Abledu, E. B. Offei, G. K. Abledu

**Affiliations:** ^1^School of Veterinary Medicine, University of Ghana, Accra, Ghana; ^2^Faculty of Applied Science and Technology, Koforidua Polytechnic, Koforidua, Ghana

## Abstract

*Background.* There is a lack of epidemiological data on musculoskeletal disorders (MSDs) among occupational drivers in Ghana. The present study seeks to estimate the prevalence, body distribution, and occupational and personal determinants of MSDs in a sample of taxi drivers in the Accra Metropolis of Ghana. *Methods*. A total of 210 participants were enrolled in this cross-sectional study. All the participants were evaluated by using a semistructured questionnaire and the standardized Nordic Musculoskeletal Disorder Questionnaire. *Results*. The estimated prevalence of MSDs was 70.5%. The prevalence of the various MSD domains was as follows: lower back pain (34.3%), upper back pain (16.7%), neck pain (15.2%), shoulder pain (11.0%), knee pain (10.0%), hip/thigh pain (2.9%), elbow pain (4.8%), ankle/feet pain (2.4%), and wrist/hand pain (1.9%). Multiple logistic regression analysis of the data showed that participants who were employee drivers, drove taxi more than 12 hours per day or at least 5 days per week, perceived their job as stressful, and were dissatisfied with their job were at a greater risk of developing MSDs. *Conclusions*. These findings call for preventive strategies and safety guidelines in order to reduce the incidence of MSDs among urban taxi drivers in Ghana.

## 1. Background

Taxis play a critical role in Ghana's urban transport system. They are typically the most accessible form of transport anytime of the day, almost anywhere, every day of the year [1] but the nature of work in the taxi industry is potentially hazardous because of the many risks involved which can vary in severity from verbal abuse, violent assault to homicide [2–5]. According to Copsey and Taylor [6], the main risks include physical risks (road accidents, vibrations, manual handling of loads, and long sitting position), chemical and biological risks (urban pollution), psychosocial risks caused by stress and violence, and risks due to individual behavior such as smoking and use of stimulants. A number of these factors are said to increase taxi drivers' risk of developing musculoskeletal disorders [5, 7–10].

Musculoskeletal disorders (MSDs) cover a wide range of conditions (e.g., tendonitis, tenosynovitis, epicondylitis, bursitis, carpal tunnel syndrome, sciatica, osteoarthrosis, myalgia, low back pain, and other idiopathic pain syndromes) that cause inflammation and degeneration of the musculoskeletal system and neurovascular structures [11]. They are a major public health problem worldwide, affect the quality of life [12], and cause substantial morbidity and disability [13] with consequent economic loss in terms of sickness absence and cost of treatment [14]. MSDs are multifactorial in terms of aetiology; the risk factors include awkward posture, manual handling, heavy lifting, strenuous tasks, and repetitive activities, while demographics, workload, and psychosocial factors are known to play parts in the pathogenesis [14].

The fact that professional driving is associated with MSDs is supported by epidemiological studies among drivers of different types of vehicle including truck drivers [15, 16], agricultural truck drivers [17], forklift drivers [18, 19], bus drivers [20], and taxi drivers [5, 7, 9, 10]. Professional taxi drivers, particularly those who work in urban cities, are said to be at increased risk of developing MSDs because of the relatively confined space in the taxi cabs as well as the long hours spent behind the wheel [9].

Despite considerable epidemiological evidence that occupational driving is associated with increased risk of MSDs, there has been no empirical research on MSDs among occupational drivers in Ghana to date. The present study, therefore, seeks to estimate the one-year prevalence, body distribution, and personal and occupational determinants of MSDs among a sample of taxi drivers in the Accra Metropolis of Ghana.

## 2. Methods

### 2.1. Participants

This cross-sectional study was conducted between November 2013 and January 2014 at various taxi ranks in Accra, the largest and capital city of Ghana. All the participants recruited in the study were male drivers with at least one year experience of driving taxi, who had no history of traumatic road or work accidents and aged 18 years or more.

### 2.2. Procedure

The participants in this study were contacted while waiting at the taxi rank. All consented participants were evaluated using a three-part semistructured questionnaire that included the standardized Nordic Musculoskeletal Questionnaire (NMQ). Participants who could neither read nor write English were assessed (at interview) using a validated and standardized translation of the questionnaire (in the local Ghanaian language). In all, a total of 300 drivers were contacted.

The first part of the questionnaire collected data on participants' sociodemographic information (age, marital status, religion, and education) and behavioural and lifestyle activities (alcohol consumption, smoking, and physical exercise). Part two of the questionnaire assessed occupational factors including taxi ownership (owner or employee driver), driving time profile (number of years of driving taxi, total driving hours per day, and number of days per week of driving), physical exposure (load handling and lifting at work), sleeping breaks in cars, and psychological factors such as perceived job stress and job satisfaction. The third part of the questionnaire mainly assessed the musculoskeletal health of drivers using the standardized Nordic Musculoskeletal Questionnaire (NMQ).

### 2.3. Nordic Musculoskeletal Questionnaire (NMQ)

The NMQ [21] is a validated [21–23] tool and consists of a general questionnaire for analysis of the point prevalence (7 days), period prevalence (12 months), and severity or disability (effect on normal activities over the last 12 months) of musculoskeletal trouble in different body areas such as neck, shoulders, elbows, wrists/hands, upper back, lower back, hips/thighs, knees, and ankles/feet. The NMQ was chosen because it is standardized, widely accepted, easy to administer and time/cost saving in terms of constructing and piloting a new questionnaire.

### 2.4. Statistical Analysis

The data were categorized and presented as proportions. Univariate logistic regression was used to obtain estimates of the prevalence odds ratio (POR) of independent factors associated with MSDs. Multiple logistic regression (simultaneous variable entry) was used to obtain estimates of the POR adjusted for the effects of all confounding variables. All statistical analyses were performed using Statistical Package for Social Sciences for Windows, version 20 (IBM Corporation, USA). Statistical significance for all tests was set at 5% alpha level.

## 3. Results

### 3.1. Response Rate, Demographics, and Personal and Occupational Factors

Out of the 300 taxi drivers contacted for the study, 45 denied participation, 26 had to terminate the study when passengers arrived, and 19 returned incomplete questionnaire, leaving 210 complete and evaluable questionnaires, indicating a response rate of 70.0%. Participants' age ranged from 23 to 61 years with mean ± standard deviation of 32.1 ± 8.7 years. The average number of years of driving taxi, driving time (in hours) per day, and driving time (in days) per week were 4.2 ± 3.3 years, 11.0 ± 2.7 hours, and 5.1 ± 1.2 days, respectively.

The sociodemographic characteristics of the subjects are presented in Table 1. In all, 63.6% (134 out of the 210) respondents were married whereas the remaining 35.4% were single; a majority of them were Christians (74.4%) as were those who had high school education (60.5%) with only 10.5% attaining above senior high school education. More than half (57.7%) of the respondents took alcoholic beverages, 6.7% smoked cigarettes, and 25.8% engaged in some form of sports activities. Generally, the respondents fell into two main categories: taxi owners (35.4%) and employee drivers (64.6%); 73.2% respondents often engaged in load handling and lifting activities at work while 29.4% took sleeping breaks in their taxis. A majority (84.7%) of the respondents perceived their job as stressful and about one-third (28.7%) of the participants were dissatisfied with their current job.

### 3.2. Musculoskeletal Disorders-NMQ

The estimated one-year prevalence of MSDs within the participants was 70.5% (i.e., 148 out of 210). The prevalence of the various MSD domains was lower back pain (72 out of 210, 34.3%), upper back pain (35 out of 210, 16.7%), neck pain (32 out of 210, 15.2%), shoulder pain (23 out of 210, 11.0%), knee pain (21 out of 210, 10.0%), hip/thigh pain (6 out of 210, 2.9%), elbow pain (10 out of 210, 4.8%), ankle/feet pain (5 out of 210, 2.4%), and wrist/hand pain (4 out of 210, 1.9%).

### 3.3. Determinants of MSDs

The association between MSDs and different personal (demographic) and occupational variables is reported in Tables 2 and 3, respectively. Lack of sports activity (OR = 3.2; 95% CI = 1.4–3.8; *P* < 0.001), being an employee driver (OR = 2.7; 95% CI = 2.0–4.2; *P* < 0.01), driving time >12 hours/day (OR = 2.6; 95% CI = 1.8–3.5; *P* < 0.01), and driving 5–7 days per week (OR = 2.9; 95% CI = 2.1–7.6; *P* < 0.001) were the significant variables that increased the odds of MSDs in the univariate analysis. Also, those who perceived their job as stressful were 3 times more likely to have MSDs as compared to those who did not (OR = 2.7; 95% CI = 1.8–3.9; *P* < 0.01), and those who were dissatisfied with their job were twice more likely to have MSDs than those who were satisfied with their job (OR = 2.3; 95% CI = 1.6–4.0; *P* < 0.05).

After adjusting for possible confounding variables in multivariate analysis, the significant factors associated with MSDs were as follows: being an employee driver, driving time >12 hours per day, driving 5–7 days per week, self-perceived job stress, and job dissatisfaction (Table 3).

## 4. Discussion

MSDs are highly prevalent among drivers, varying between 53% and 91% in different parts of the world [5, 15, 24–26]. However, there has been no empirical study on MSDs among occupational drivers in Ghana to date, hence the need for the present study. The estimated period prevalence of MSDs (70.5%) in this study is consistent with the worldwide prevalence range and the hypothesis that urban taxi drivers are at increased risk of developing MSDs. Also, the observed prevalence and pattern of distribution of MSDs in the different body regions suggest a somewhat degree of diversity in the musculoskeletal pain reported by the participants. This diversity could, in part, be due to individual differences in the modulators of self-reported pain which include genetic composition, psychological status, and economic and sociocultural factors [27].

Low back pain (LBP) was the commonest symptom, occurring in 34.3% of the drivers. Whereas this prevalence rate is lower than the prevalence range (45.8%–59%) reported among Caucasians [5, 7, 9, 28], it is similar to the 30.66% rate reported among Nigerian taxicab drivers with similar sociocultural characteristics [29].

Increased odds of developing MSDs with increased duration of driving were observed among the participants in this study (i.e., driving >12 hours/day and at least 5 days/week was significantly associated with increased risk of MSDs). Prolonged driving (sitting) or static posture in a relatively confined and vibrating taxicab environment (that offers little room for flexing and movement of the limbs) can cause postural strain on the musculoskeletal system. This can be uncomfortable and stressing and thus increase susceptibility to musculoskeletal injury/pain. An association between MSDs and daily or weekly driving duration is supported by several studies. For instance, a study [10] among taxi drivers in Taipei city of Taiwan found that driving more than 10 hours/day increased the risk of knee pain in this population. Chen et al. [9] also found that driving taxi more than 4 hours/day was associated with a greater risk of low back pain. Driving >71 hours/week [5] and >20 hours/week [24] was also found to be associated with high frequency of low back pain.

From this study, employee drivers were more likely to develop MSDs than owner drivers presumably because they (employee-drivers) have less control over their working hours. Contrary to reports in the literature [5, 9, 30] age, number of years of driving, alcohol consumption, smoking, taking sleeping breaks in the taxi, and work-related activities such as load handling and lifting at work were not associated with MSDs in the present study. This could be due to methodological, sampling, or population differences.

In this study, psychosocial factors such as self-perceived job stress and job dissatisfaction were significantly associated with MSDs even after adjusting for possible confounders in multivariate analysis. The exact role of psychosocial factors in the development of MSDs is poorly understood; however, they are known to cause psychosocial stress. Psychosocial stress can cause muscle tension, mechanical strain of the spinal cord, and fatigue, all of which could lead to traumatic injury [30], hence MSDs. Several studies [9, 10] have identified self-perceived job stress, job dissatisfaction, and mental health as important determinants of musculoskeletal discomfort of urban taxi drivers.

Lack of sports activities was associated with increased odds of MSDs in this study; however, this observation diminished after adjusting for potential confounders including driving duration, ownership of taxi, self-perceived job stress, and job dissatisfaction. Randomized clinical trials have shown that physical activity has direct effect on physical fitness [31]. That said, regular physical activity (according to advocates in USA) would prevent communicable diseases, decrease all-cause mortality, and promote health [32]. However, whether physical activity is beneficial to musculoskeletal health is unclear. Some studies [13, 33] have indicated favourable relation between the frequency of leisure time sports activity and MSDs; others [32, 34] found no association between the duo while Miranda et al. [35] reported adverse effects. In their study, Miranda et al. [35] found that individuals who engaged in active walking had more sciatic pain; active volleyball players had more shoulder pain while those who actively practiced trekking had more knee pain than those who practiced these activities less. Physical activity-related injuries are common and it is possible that these injuries were included in self-reported MSDs in this study. Randomized clinical trials on the effects of physical activity on MSDs are required.

## 5. Conclusions

The estimated one-year prevalence of MSDs within the taxi drivers was 70.5%. Taxi drivers have a high prevalence of MSDs that is dependent on ownership of taxi, daily and weekly driving duration, self-perception of job stress, and job satisfaction. These findings call for preventive strategies and safety guidelines in order to reduce the incidence of MSDs of urban taxi drivers in Ghana. This cross-sectional study provides the baseline for elaborative studies in the future.

## Figures and Tables

**Table 1:** Sociodemographic characteristics of the participants (*n* = 210).

Characteristic	*n*	%
Marital status		
Married	134	63.6
Single	76	36.4
Education		
Below senior high school	61	28.9
Senior high school	127	60.5
Above senior high school	22	10.5
Religion		
Christian	156	74.4
Moslem	45	21.5
Others	9	4.1
Taxi ownership		
Owner	74	35.4
Employee	136	64.6
Perceived job stress		
None	32	15.3
Moderate to severe	178	84.7
Sleeping breaks in car		
Yes	62	29.4
No	148	70.6
Job satisfaction		
Satisfied	150	71.3
Dissatisfied	60	28.7
Alcohol		
Yes	89	42.3
No	121	57.7
Smoking		
Yes	14	6.7
No	196	93.3
Regular sports activity		
yes	54	25.8
No	156	74.2
Load handling and lifting at work		
Yes	154	73.2
No	56	26.8

Percentages may not sum up to exactly 100 because of approximations.

**Table 2:** Personal factors associated with MSDs among the participants (*n* = 210).

Parameter	OR (95% CI)	aOR (95% CI)

Age (years)		
<45	0.6 (0.2–2.2)	—
46–55	1	—
>55	0.7 (0.4–2.8)	—
Marital status		
Married	1	—
Single	2.3 (1.9–4.8)	—
Education		
Below senior high school	0.4 (0.1–1.9)	—
Senior high school	1	—
Above senior high school	0.5 (0.2–2.1)	—
Religion		
Christian	2.3 (1.7–4.3)	—
Islam	2.6 (1.8–4.5)	—
Others	1	—
Alcohol		
No	1	—
Yes	2.7 (2.1–3.5)	—
Smoking		
No	1	—
Yes	0.8 (0.1–3.9)	—
Regular sports activity		
No	3.2 (1.4–3.8)∗∗∗	0.8 (0.3–1.9)
Yes	1	1

OR: odds ratio; aOR: adjusted odds ratio; CI: confidence interval; ^***^ *P* < 0.001.

**Table 3:** Occupational factors associated with MSDs among the participants (*n* = 210).

Parameter	OR (95% CI)	aOR (95% CI)
Type of taxi driver		
Owner	1	1
Employee	2.7 (2.0–4.2)∗∗	2.4 (1.9–3.6)∗
Number of years of driving		
<6	0.4 (0.1–2.7)	—
6–10	1	—
>10	0.7 (0.2–2.1)	—
Driving time per day (hours)		
<10	0.5 (0.1–1.9)	0.3 (0.1–1.2)
10–12	1	1
>12	2.6 (1.8–3.5)∗∗	1.7 (1.1–2.2)∗
Driving time per week (days)		
<5	1	1
5–7	2.9 (2.1–7.6)∗∗∗	2.1 (1.5–4.1)∗∗∗
Perceived job stress		
None	1	1
Moderate to severe	2.7 (1.8–3.9)∗∗	2.3 (1.5–3.0)∗∗
Sleeping breaks in car		
No	1	—
Yes	2.4 (1.8–11.9)	—
Job satisfaction		
Satisfied	1	1
Dissatisfied	2.3 (1.6–4.0)∗	2.0 (1.3–3.6)∗
Load handling and lifting at work		
No	1	1
Yes	2.4 (1.4–5.7)	—

OR: odds ratio; aOR: adjusted odds ratio; CI: confidence interval; ^*^ *P* < 0.05; ^**^ *P* < 0.01; ^***^ *P* < 0.001.
